# Management and long-term outcomes of acute right colonic diverticulitis and risk factors of recurrence

**DOI:** 10.1186/s12893-022-01578-z

**Published:** 2022-04-07

**Authors:** Zhilong Ma, Weiwei Liu, Jia Zhou, Le Yao, Wangcheng Xie, Mingqi Su, Jin Yang, Jun Shao, Ji Chen

**Affiliations:** 1 Department of General Surgery, Tongren Hospital, Shanghai Jiao Tong University School of Medicine, Shanghai, 200336 China; 2 Department of Gastroenterology, Tongren Hospital, Shanghai Jiao Tong University School of Medicine, Shanghai, 200336 China; 3 Department of General Surgery, Shanghai Tenth People’s Hospital, Tongji University School of Medicine, Shanghai, 200072 China

**Keywords:** Acute Right Colon Diverticulitis, Conservative treatment, Surgical treatment, Recurrence

## Abstract

**Background:**

Acute right-sided colonic diverticulitis (RCD) is a common disease in Asian populations for which the optimal treatment remains controversial. The aim of this study was to investigate management and evaluate long-term outcomes of treatment in patients with acute RCD.

**Methods:**

We retrospectively collected and analyzed clinical data for patients with acute RCD admitted to the Tongren Hospital, Shanghai Jiao Tong University School of Medicine from December 2015 to December 2020. The patients were divided into two groups, according to primary treatment strategy, which was either conservative treatment or surgical treatment.

**Results:**

A total of 162 consecutive patients with acute RCD were enrolled in the study. There was no significant difference in age, sex, history of abdominal surgery, medical co-morbidities, fever, previous history of RCD, treatment success rate and incidence of complications between the conservative and surgery groups. However, the recurrence rate in conservative groups was significantly higher than in surgery groups (16.53% vs 2.44%, *P* = 0.020). And more frequent bowel movements and previous history of RCD increased the risk of recurrence of acute RCD. Moreover, there was no significant difference in either treatment success rate or the overall recurrence rate between the patients with uncomplicated diverticulitis and patients with complicated diverticulitis.

**Conclusions:**

Surgical treatment is also safe and effective for acute RCD. Surgical treatment should mainly be considered for patients with acute RCD with recurrence risk factors (more frequent bowel movements and previous history of RCD) or with complicated acute RCD.

## Introduction

Acute colonic diverticulitis is a common gastrointestinal disease in both outpatient and inpatient settings [1]. Although acute right-sided colonic diverticulitis (RCD) accounts for only 1–5% of acute diverticulitis in Western countries, acute RCD is more prevalent in Asian populations and accounts for 55–98% of colonic diverticulitis cases [2–7]. Acute RCD is more commonly occurred in younger age groups and clinical course is mild [8], while Left-sided colonic diverticulitis is strongly associated with increasing age and increased intraluminal pressure [6]. Acute RCD may thus represent a disease process that is different from its left-sided counterpart and its etiology may involve congenital or genetic factors, which lead to true diverticula [9]. At present, there are no specific guidelines for the management of acute RCD and treatment methods are typically based on previous treatment experience with left-sided disease [10, 11].

Conservative treatment with intravenous antibiotics and bowel rest is usually recommended for the management of acute uncomplicated RCD in Asia [12–15]. Nevertheless, some patients are at risk of exacerbation and emergent surgery need to be performed, in which case right colectomy could be required. Furthermore, about 6.8–20.5% of cases with RCD develop recurrence after conservative management [12, 16, 17]. Thus, conservative treatment may thus not be the best option for every patient with acute RCD but there is no definitive treatment strategy for acute RCD.

The purpose of this study was to investigate management and evaluate long-term outcomes of treatment in patients with acute RCD, and explore risk factors for recurrence of RCD.

## Materials and methods

### Patients and data collection

This retrospective study involved a cohort of 162 patients (age ≥ 18 years), treated at the Department of General Surgery, Tongren Hospital, Shanghai Jiao Tong University School of Medicine from December 2015 to December 2020. Acute RCD was defined according to previous studies, as the presence of colonic diverticular disease with localized colonic wall thickening and/or stranding of pericolonic fat [1, 11, 18]. A diagnosis of acute RCD was confirmed by abdominal ultrasonography (US), computed tomography (CT), colonoscopy, and further clinical assessments. Furthermore, acute RCD was also diagnosed by postoperative histological examination. Patients with missing data on length of stay or disposition on discharge were excluded from the study, as were patients with a concomitant diagnosis of colorectal cancer, inflammatory bowel disease (IBD), bowel ischemia, acute appendicitis and gastrointestinal hemorrhage. Other exclusion criteria included hemodynamic instability, multi-organ failure, and American Society of Anesthesiologists (ASA) classification IV or higher. The clinical data, including demographic information, medical history, symptoms, morbidity, mortality, and follow-up information were collected and retrospectively analyzed. The study protocol was approved by the Institutional Review Board of Tongren Hospital, Shanghai Jiao Tong University School of Medicine. Informed consent was waived due to the retrospective nature of this study and data were analyzed anonymously.

### Treatment characteristics

Each patient received conservative or surgical treatment, based on his or her evaluation and the severity of the acute RCD. Conservative treatment included bowel rest and intravenous antibiotics, followed by a week of oral antibiotics after discharge. When the patient has no fever and abdominal pain, oral intake was resumed. Failure of conservative treatment led to surgical treatment. Emergent surgery was executed within 12 h after admission, or failure of conservative treatment led to emergent surgery, elective surgery for patients suffering from recurrent diverticulitis. Surgical procedures including appendectomy with postoperative antibiotic treatment, diverticulectomy, diverticulectomy with appendectomy, and right hemicolectomy/ileocecal resection.

### Outcome definitions and follow-up

**Fig. 1:**
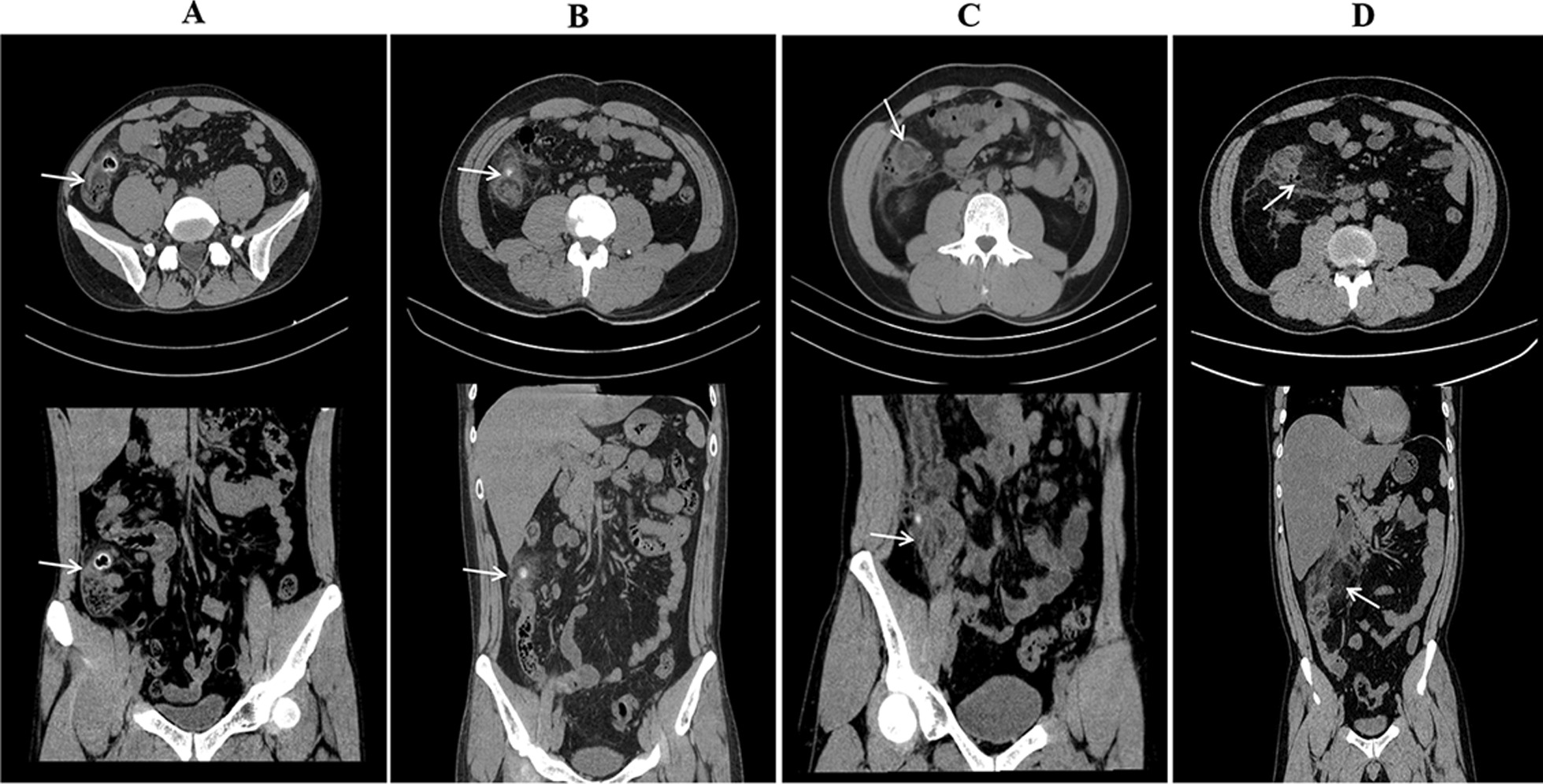
**A** Hinchey stage I diverticulitis: Arrow points to the inflamed cecal diverticulum with thickening of the colonic wall of the cecum, the patient received conservative. **B** Hinchey stage II diverticulitis: Arrow points to inflamed mass, the patient was executed diverticulectomy with appendectomy. **C** Hinchey stage III diverticulitis: Arrow points to localized abscess, the patient was executed ileocecal resection. **D** Hinchey stage IV diverticulitis: Arrow points to free air and ruptured abscess, the patient underwent emergency right hemicolectomy

Patients were categorized according to the Hinchey classification by evaluating their radiological images [19]. The classifications were Grade I: inflamed diverticulum; Grade II: inflamed mass; Grade III: localized abscess/fistula; and Grade IV: perforation/ruptured abscess with generalized peritonitis (Fig. 1). Grade I signifies uncomplicated diverticulitis, which involves thickening of the colon wall and pericolonic inflammatory changes, whereas Grades II, III, and IV indicate complicated diverticulitis, which also includes abscess, peritonitis, obstruction, and/or fistulae. Mortality was defined as in-hospital death.

All cases were followed up for at least 12 months. Outpatient visits were executed at 1 week, 1 month, 6 months, and 12 months after discharge. Telephone follow-up was conducted every six months after 12 months. During this follow-up period, if the patient has signs or symptoms indicating the recurrence of RCD, the patient will be recalled for CT scan. The primary endpoint was recurrence, based on CT evidence of recurrent RCD at 1 month after discharge. Recurrence within 1 month of discharge was regarded as treatment failure [13]. The secondary endpoints included treatment success, death and complications. Complications were classified using the Clavien-Dindo classification [20], which is based on the therapy required to treat them.

### Statistical analysis

All data analyses were performed using IBM SPSS statistics for Windows, version 22.0. (IBM Corporation, Armonk, NY, USA). Continuous values are presented as mean ± standard deviation (SD). Statistical analysis was carried out using the independent sample t-test or Mann–Whitney U test for continuous data, and the Pearson’s χ2 test for categorical data. A probability (*P*) value of < 0.05 was considered to be statistically significant.

## Results

**Fig. 2:**
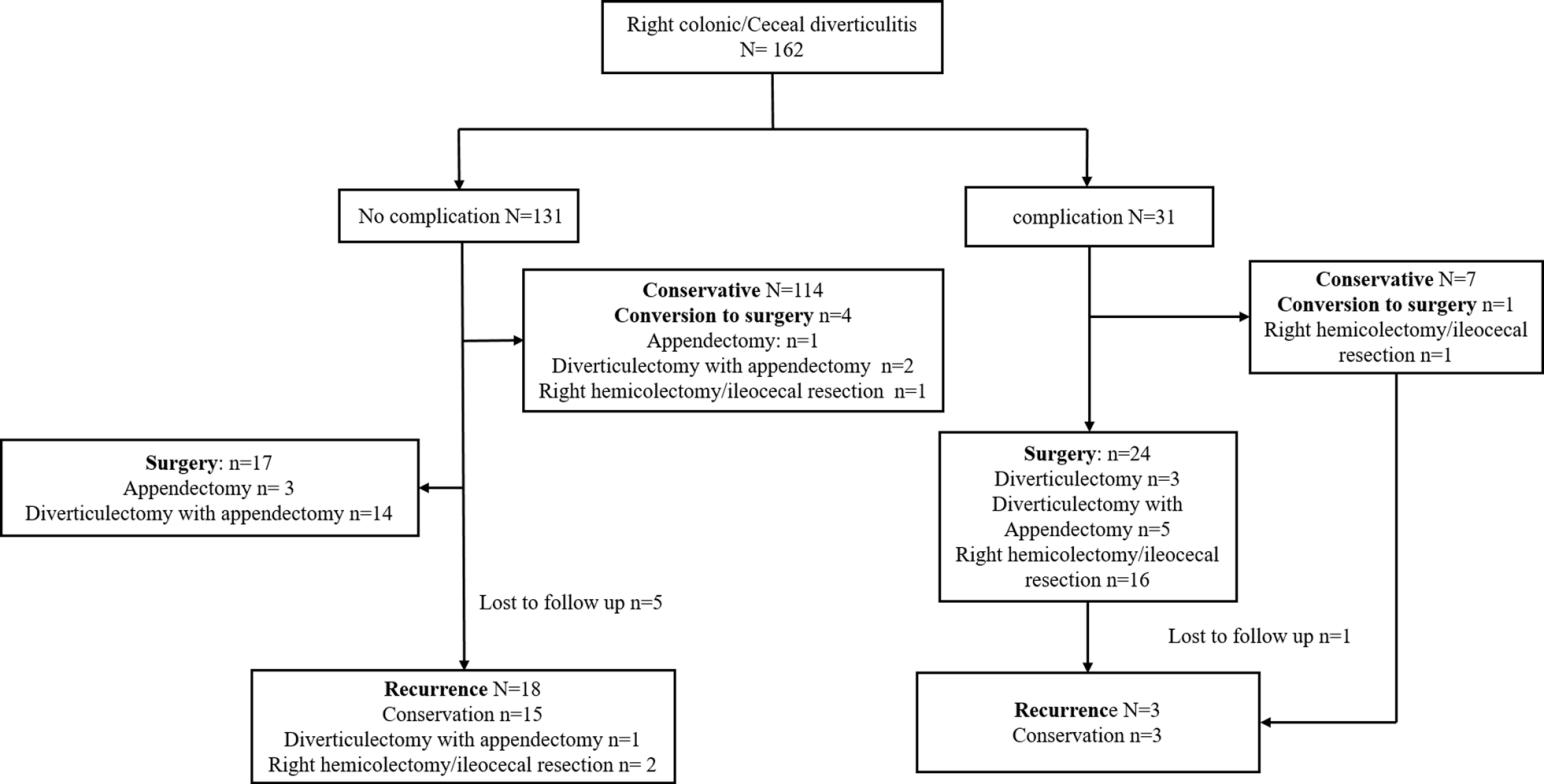
Flow and outcomes of study patients

A total of 162 consecutive patients with acute RCD, treated between December 2015 and December 2020, were enrolled in this retrospective study (Fig. 2). All patients with acute RCD were divided into two groups according to primary treatment strategy, a conservative treatment group (n = 121) and a surgery treatment group (n = 41) (Table 1). There was no significant difference in terms of age, sex, body-mass index (BMI), current smoking habits, ASA status, previous history of abdominal surgery, medical co-morbidities, fever, and previous history of RCD between the two groups. Levels of c-reactive protein (CRP) and procalcitonin were not statistically different between the two groups, whereas numbers of white blood cells (WBC) and neutrophils were significantly higher in the surgery group than in the conservative treatment group. The Hinchey Stage was also significantly different between the two groups (*P* < 0.001), patients with complicated disease were more likely to be considered for surgical treatment.

**Table 1:** Baseline characteristics of patients

Characteristics	Conservation (n = 121)	Surgery (n = 41)	*P*
Age (year)	55.00 (39.50, 63.00)	47.00 (28.50, 63.50)	0.147
Gender male, n (%)	74 (61.16%)	29 (70.73%)	0.271
BMI (kg/m^2^)	22.40 (20.80, 24.20)	23.10 (21.50, 24.15)	0.328
Current smoking, n (%)	12 (9.92%)	6 (14.63%)	0.587
ASA, n (%)			0.642
I	99 (81.82%)	31 (75.61%)	
II	14 (11.57%)	7 (17.07%)	
III	8 (6.61%)	3 (7.31%)	
Previous abdominal surgery, n (%)	31 (25.62%)	7 (17.07%)	0.264
Appendectomy	13 (10.74%)	4 (9.76%)	1.000
Others	18 (14.88%)	3 (7.31%)	0.213
Medical co-morbidity, n (%)	65 (53.72%)	15 (36.59%)	0.058
Ischemic heart disease or heart failure	11 (9.09%)	1 (2.43%)	0.289
Pulmonary tuberculosis history	1 (0.83%)	2 (4.88%)	0.158
Chronic obstructive pulmonary disease	3 (2.48%)	2 (4.88%)	0.806
Diabetes mellitus	7 (5.78%)	1 (2.44%)	0.662
Hypertension	29 (23.97%)	6 (14.63%)	0.053
Chronic kidney disease	2 (1.65%)	0 (0%)	1.000
Anti-platelet medication	3 (2.48%)	2 (4.88%)	0.806
Others	7 (5.79%)	3 (7.32%)	1.000
Fever, n (%)	34 (28.10%)	14 (34.15%)	0.464
Lab			
WBC	11.07 ± 3.19	12.51 ± 4.13	0.047
Neu	8.37 (6.50, 10.62)	10.27 (7.15, 12.51)	0.015
CRP	51.21 (21.00, 91.25)	69.00 (19.45, 86.25)	0.469
PCT	0.01 (0.01, 0.09)	0.01 (0.01, 0.29)	0.265
Hinchey Stage, n (%)			< 0.001
I	114 (94.21%)	17 (41.46%)	
II	4 (3.31%)	1 (2.44%)	
III	2 (1.65%)	2 (4.88%)	
IV	1 (0.83%)	21 (51.22%)	
Complicated diverticulitis, n (%)	7 (5.79%)	24 (58.54%)	< 0.001
Previous history of RCD, n (%)	11 (9.09%)	2 (4.88%)	0.599
Follow-up, months	24.00 (9.50, 35.50)	27.00 (7.00, 42.00)	0.883

In the conservative treatment group, 116 patients (95.87%) were successfully treated with bowel rest and intravenous antibiotics, while five patients (4.13%) required conversion to surgery and were considered to be treatment failures (Table 2). In the surgical treatment group, 40 patients (97.56%) were treated successfully. However, there was not statistically different in treatment success rate or mortality between the two groups. The incidence of complications in the surgical treatment group was not significantly higher than that in the conservative treatment group (14.63% vs 5.79%, *P* = 0.142). Moreover, the length of postoperative hospital stay was significantly longer in the surgical treatment group than in the conservative treatment group (7 d vs 5 d, *P* < 0.001). Notably, the recurrence rate in patients with conservative treatment was significantly higher than that in patients with surgical treatment group (16.53% vs 2.44%, *P* < 0.020). And most patients who suffered a recurrence continued to be treated successfully without surgery. Taken together, these results demonstrated that both conservative and surgical treatment of acute RCD were safe and effective.

**Table 2:** Operation outcome data

Characteristics	Conversation (n = 121)	Surgery (n = 41)	*P*
Treatment success, n (%)	116 (95.87%)	40 (97.56%)	0.986
Conversion to surgery	5	–	
Appendectomy with postoperative antibiotic treatment	1	–	
Diverticulectomy with appendectomy	2	–	
Diverticulectomy	0	–	
Right hemicolectomy/ileocecal resection	2	–	
Operation time, min	90.00 (82.00, 100.00)	105.00 (60.00, 142.00)	0.818
Any complication, n (%)	7 (5.79%)	6 (14.63%)	0.142
Abscess and worsening colitis	0 (0%)	1 (2.44%)	0.253
Spreading peritonitis	2 (1.65%)	0 (0%)	1
Fecal peritonitis	0	0	–
Wound infection	0 (0%)	2 (4.88%)	0.063
Bowel injury	0 (0%)	0 (0%)	–
Paralytic ileus	1 (0.83%)	0 (0%)	1.000
Surgical sites bleeding	0	0	–
Acute kidney injury	0	0	–
Urinary tract infection	0 (0%)	1 (2.44%)	0.253
Pneumonia	4 (3.31%)	2 (4.88%)	1.000
Mortality, n (%)	0 (0%)	0 (0%)	–
Postoperative hospital stays, days	5.00 (3.00, 6.00)	7.00 (5.00, 10.00)	< 0.001
Recurrence, n (%)	20 (16.53%)	1 (2.44%)	0.020

Although both our own study and previous studies [14, 21] showed that conservative treatment of acute RCD was safe and effective, it remains unclear whether or not patients with uncomplicated and complicated RCD are both recommended for the same treatment. All patients were, therefore, divided into either a uncomplicated group or a complicated group, according to their radiological imaging features and clinical assessment. Out of 131 patients with uncomplicated diverticulitis, a total of four (3.05%) of the conservatively treated patients later underwent surgery (Table 3). Despite 24 (77.42%) patients had undergone surgery in complicated group, there was no significant difference in either treatment success rate or mortality between the two groups. However, the incidence of complications in the complications group was significantly higher than that in the complications-free group (*P* = 0.003). Furthermore, the length of postoperative hospital stay in the complications group was significantly longer than in the complications-free group (8 d vs 5 d, *P* < 0.001). However, there was no significant difference in the overall recurrence rate between the two groups (13.74% vs 9.68%, *P* = 0.758). Therefore, based on patient evaluation and severity of acute RCD, complicated RCD patients should thus be recommended for early emergency surgery.

**Table 3:** Severity and management of complicated diverticulitis

n = 162	Uncomplicated (n = 131)	Complicated (n = 31)			*P*
	I (n = 131)	II (n = 5)	III (n = 4)	IV (n = 22)	
First Treatment Type					< 0.001
Conservation, n (%)	114 (87.02%)	7 (22.58)			
Conversion to surgery, n (%)	4 (3.5%)	1 (14.3%)			
Surgery, n (%)	17 (12.98%)	24 (77.42%)			
Experienced Surgery, n (%)	21 (16.03%)	25 (80.65%)			< 0.001
Appendectomy, n (%)	4 (3.05%)	0 (0%)			1.000
Diverticulectomy with appendectomy, n (%)	16 (12.21%)	5 (16.13%)			0.775
Diverticulectomy, n (%)	0 (0%)	3 (9.68%)			0.006
Right hemicolectomy/ileocecal resection, n (%)	1 (0.76%)	17 (54.84%)			< 0.001
Success, n (%)	126 (96.18%)	30 (96.77%)			1.000
Any complication, n (%)	6 (4.58%)	7 (22.58%)			0.003
Abscess and worsening colitis, n (%)	1 (0.76%)	0 (0%)			1.000
Spreading peritonitis, n (%)	1 (0.76%)	1 (3.23%)			0.347
Fecal peritonitis, n (%)	0	0			–
Wound infection, n (%)	0 (0%)	2 (6.45%)			0.036
Bowel injury, n (%)	0 (0%)	0 (0%)			–
Paralytic ileus, n (%)	1 (0.76%)	0 (0%)			1.000
Surgical sites bleeding, n (%)	0	0			–
Acute kidney injury, n (%)	0	0			–
Urinary tract infection, n (%)	0 (0%)	1 (3.23%)			0.191
Pneumonia, n (%)	3 (2.29%)	3 (9.68%)			0.153
Mortality, n (%)	0 (0%)	0 (0%)			–
Length of post-op stay, day	5.00 (3.00, 6.00)	8.00 (7.00,12.00)			< 0.001
Recurrence, n (%)	18 (13.74%)	3 (9.68%)			0.758
Time of follow-up, month	24.00 (9.00, 36.25)	28.50 (9.75, 43.25)			0.472

A total of 162 patients with RCD were evaluated during the study period, with a mean follow-up period of 24.9 months. The overall recurrence rate of RCD was 12.96%. Among these recurrences, 18 patients continued to have successful non-operative therapy, two patients had complications and were treated by right hemicolectomy/ileocecal resection, and one patients were treated by diverticulectomy with appendectomy. There was no statistically significant intergroup difference in sex, age, BMI, previous abdominal surgery, medical co-morbidities, current smoking habits, fever and laboratory tests (Table 4). The recurrence rate in patients with complicated RCD was not significantly higher than that in patients with uncomplicated diverticulitis (*P* = 0.758). Moreover, despite the recurrence rate in patients with conservative treatment was significantly higher than that in patients with surgical treatment group, the recurrence rate was not affected by operation time, postoperative complications or length of postoperative hospital stay. Remarkably, in our study we found that the recurrence rate in patients with previous history of RCD was significantly higher than that in patients suffering a first attack of diverticulitis (*P* < 0.001). Furthermore, more frequent bowel movements also increased the risk of recurrence of acute RCD (*P* < 0.001).

**Table 4:** Demographic and clinical outcomes of the study population

	No Recurrence (n = 141)	Recurrence (n = 21)	*P*
Age (year)			0.893
< 40	40 (28.37%)	5 (23.81%)	
40–70	90 (63.83%)	14 (66.67%)	
> 70	11(7.80%)	2 (12.52%)	
Gender male, n (%)	90 (63.83%)	13 (61.90%)	0.864
BMI (kg/m^2^)	22.60 (21.00, 24.20)	23.50 (21.50, 24.20)	0.505
Current smoking, n (%)	15 (10.64%)	3 (14.29%)	0.901
ASA			0.786
I	113 (80.14%)	17 (80.95%)	
II	19 (13.48%)	2 (9.52%)	
III	9 (6.38%)	2 (9.52%)	
Previous abdominal surgery, n (%)	33 (24.40%)	5 (23.8%)	0.814
Appendectomy	16 (11.35%)	1 (4.76%)	0.591
Others	17 (12.06%)	4 (19.05%)	0.588
Medical co-morbidity, n (%)	70 (49.65%)	10 (47.62%)	0.862
Ischemic heart disease or heart failure	10 (7.09%)	2 (9.52%)	1.000
Pulmonary tuberculosis history	2 (1.42%)	1 (4.76%)	0.342
Chronic obstructive pulmonary disease	4 (2.84%)	1 (4.76%)	0.505
Diabetes mellitus	8 (5.67%)	0 (0%)	0.562
Hypertension	31 (21.98%)	4 (19.05%)	0.983
Chronic kidney disease	2 (1.42%)	0 (0%)	1.000
Anti-platelet medication	4 (2.84%)	1 (4.76%)	0.505
Others	9 (6.38%)	1 (4.76%)	1.000
Frequency of Bowel Movements			< 0.001
More than once daily	9 (6.38%)	11 (52.38%)	< 0.001
Daily	124 (87.94%)	7 (33.33%)	< 0.001
Every 2 days	4 (2.84%)	1 (4.76%)	0.505
Every 3–4 days or less frequently	4 (2.84%)	2 (9.52%)	0.174
Fever, n (%)	42 (29.79%)	6 (28.57%)	0.909
Lab			
WBC	11.29 ± 3.27	12.67 ± 4.92	0.262
Neu	8.56 (6.53, 10.99)	9.45 (7.51, 12.46)	0.324
CRP	54.35 (21.15, 89.11)	73.54 (18.29, 124.35)	0.615
PCT	0.01 (0.01, 0.10)	0.01 (0.01, 0.41)	0.902
Complicated diverticulitis, n (%)	28 (19.86%)	3 (14.29%)	0.758
Previous history of RCD, n (%)	3 (2.12%)	10 (47.62%)	< 0.001
First Treatment Type			0.010
Conservation	96 (68.09%)	20 (95.24%)	
Surgery	45 (31.91%)	1 (4.76%)	
Experienced Surgery, n (%)	45 (31.91%)	1 (4.76%)	0.010
Appendectomy, n (%)	3 (2.13%)	1 (4.76%)	0.429
Diverticulectomy with appendectomy, n (%)	21 (14.89%)	0 (0%)	0.122
Diverticulectomy, n (%)	3 (2.13%)	0 (0%)	1.000
Right hemicolectomy/ileocecal resection, n (%)	18 (12.77%)	0 (0%)	0.172
Operation time, min	102.50 (60.00, 146.25)	82.5 (42.75, 120.00)	0.418
Any complication, n (%)	9 (6.38%)	1 (4.76%)	1.000
Abscess and worsening colitis, n (%)	1 (0.71%)	0 (0%)	1.000
Spreading peritonitis, n (%)	1 (0.71%)	1 (4.76%)	0.243
Fecal peritonitis, n (%)	0 (0%)	0 (0%)	–
Wound infection, n (%)	2 (1.42%)	0 (0%)	1.000
Bowel injury, n (%)	0 (0%)	0 (0%)	–
Paralytic ileus, n (%)	1 (0.71%)	0 (0%)	1.000
Surgical sites bleeding, n (%)	0 (0%)	0 (0%)	–
Acute kidney injury, n (%)	0 (0%)	0 (0%)	–
Urinary tract infection, n (%)	1 (0.71%)	0 (0%)	1.000
Pneumonia, n (%)	3 (2.13%)	0 (0%)	1.000
Length of post-op stay, day	5.00 (4.00, 7.00)	5.00 (3.00, 7.00)	0.220

## Discussion

The incidence of acute diverticulitis is increasing worldwide and caused a growing burden of socioeconomic [1, 22, 23]. Although it is recognized that RCD is more common in Eastern than in Western countries, the management of RCD remains controversial. According to guidelines or suggestions for the management of acute left colonic diverticulitis [11, 24], the patients with Hinchey I disease were recommended antibiotic therapy; the patients with Hinchey II disease (a larger abscess) should be drained percutaneously; the patients with Hinchey III and IV disease, the first choice remains emergency surgery; elective surgery should be considered in patients with a high risk of recurrence. In past decades, there has been a strong tendency towards more conservative management of acute RCD [21, 25]. A study by Destek et al*.* showed that conservative management of uncomplicated RCD with antibiotics is a safe and effective treatment option [14]. Recently, a multicenter retrospective study also showed that conservative treatment can be used safely and effectively for uncomplicated RCD, with a low recurrence rate of 6.8% [12].

In our study, 116 patients (95.87%) were successfully treated with bowel rest and intravenous antibiotics, while the 40 patients (97.56%) were treated successfully by surgery. Despite there was not statistically different in treatment success rate between the two groups, the recurrence rate in patients with conservative treatment was significantly higher than that in patients with surgical treatment group. Therefore, although conservative treatment is beneficial for patients with RCD, there remains the possibility of recurrence and patients should receive individualized treatment, according to their different risk of recurrence of RCD.

So far, however, the risk factors for recurrent RCD remain unclear. Park et al*.* found that the recurrence rate following conservative treatment of acute uncomplicated RCD was 15.8% after the first attack, and that the risk of recurrence was high in patients with multiple diverticula and intraperitoneally located diverticulitis [17]. A recent systematic review showed that acute colonic diverticulitis with abscess formation, a young age and previous recurrences all increased the risk of recurrence [26]. Kim et al*.* also showed that disease recurred in 10.47% of patients with acute RCD after non-surgical treatment; the risk factors of recurrence were smoking and long hospital stay [27]. In our own study, we found that more frequent bowel movements and previous history of RCD increased the risk of recurrence of acute RCD. Consequently, for RCD patients with recurrence risk factors, the possibility of recurrence was high and surgical treatment should be recommended.

The optimal choice of surgery for acute RCD, however, remains no clear, especially when RCD is confirmed intraoperatively. Options range from open to minimally invasive surgery, from diverticulectomy to colonic resection with primary anastomosis. Previous a single Western center study revealed that diverticulectomy is feasible and valid for RCD, and complication rate is low [28]. Laparoscopic diverticulectomy (LD) is a minimally invasive surgical procedure, with a low complication rate compared to right colectomy [23, 29]. A recent study showed that LD is safe and effective in patients who are concerned about disease progression and recurrence [13]. Furthermore, previous studies revealed that colectomy is executed when complications occur or when malignancy is strongly suspected [30, 31]. In our study, based on patient’s evaluation and the severity of the acute RCD, 97.56% of cases were successfully treated by surgery, included of 22 patients (53.66%) were successfully treated by diverticulectomy or diverticulectomy with appendectomy. Despite four patients underwent appendectomy, possibly because of a presumptive clinical diagnosis of acute appendicitis, without diagnostic images, the overall the recurrence rate was very low (2.44%).

Although US and CT now provide high specificity and sensitivity in the diagnosis of acute RCD [32, 33], the differential diagnosis between acute RCD and acute appendicitis is challenging [34, 35]. Acute appendicitis is the most common false diagnosis for cecum diverticulitis [36] and the two cannot be distinguished clinically. More than 70% of cecum diverticulitis cases have a false diagnosis of acute appendicitis and, when the patient is taken into surgery, the surgeon is confronted with a healthy appendix and cecum diverticulitis [37]. It was recently recommended that diverticulectomy and appendectomy were adequate treatments for solitary cecum diverticulitis when inflammation was not so severe [38]. Furthermore, a recent review found that, in cases of operative but conservative treatment for solitary cecum diverticulitis, appendectomy could be justified to avoid misdiagnosis in case of future episodes of solitary cecum diverticulitis [39]. In our study, 4 patients (2.47%) underwent appendectomy, the treatment success rate was 100%, although one patients occurred recurrence after 12 months. Taken together, appendectomy is also recommended for patients for whom appendicitis cannot be completely excluded.

There were several potential limitations to the present study, including its retrospective design, significant selection biases, and small sample size in a single center. Treatments for patients with acute RCD included in the study were heterogeneous because experts in the center used different treatment options, based on patients’ symptoms, clinical findings, and their own experience. Moreover, this is also confused for considering both emergency and elective surgery for uncomplicated acute RCD. Risk factors for recurrence of RCD, including dietary habits, physical activity, and defecation habits, were not included in our study, which meant that the results of our study could not be generalized to the entire population. Despite an outpatient follow-up period of more than 2 years, the study still lacked long-term follow-up data to investigate long-term complications and morbidity. We will address these limitations in future studies.

In conclusion, surgical treatment and conservative treatment methods are both safe and effective for acute RCD. However, surgical treatment should mainly be considered for patients with acute RCD with recurrence risk factors (more frequent bowel movements and previous history of RCD) or with complicated acute RCD.

## Data Availability

All patient data and clinical images adopted are contained in the medical files of Tongren Hospital, Shanghai Jiao Tong University School of Medicine. The data supporting the conclusions of this article are included within the article and its figures.

## Abbreviation

RCD: Right-sided colonic diverticulitis
